# Supplementary material for: Co-Carbonized Waste Polythene/Sugarcane Bagasse Nanocomposite for Aqueous Environmental Remediation Applications
Source: Nanomaterials (Basel). 2023 Mar 27;13(7):1193. doi: 10.3390/nano13071193 (PMC10097173; doi:10.3390/nano13071193)
Supplement: Supplementary file 1 [file nanomaterials-13-01193-s001.zip › nanomaterials-2288013-supplementary.pdf]

Supplementary Information

Co-carbonized Waste Polythene/Sugarcane Bagasse Nanocomposite for Aqueous Environmental Remediation Applications

Moonis Ali Khan [1,*], Ayoub Abdullah Alqadami [1], Saikh Mohammad Wabaidur [1] and Byong-Hun Jeon [2]

[1] Chemistry Department, College of Science, King Saud University, Riyadh 11451, Saudi Arabia
[2] Department of Earth Resources and Environmental Engineering, Hanyang University, Seoul 04763, the Republic of Korea
* Correspondence: mokhan@ksu.edu.sa or moonisalikhan@gmail.com

Table of contents

**Index**	**Caption**
Text S1	Adsorption isotherm models used.
Text S2	Adsorption kinetic models used.
Figure S1	TEM images of SBPE (a), and SBPEAC (b) composites.
Figure S2	Non-linear isotherm models for MG adsorption on SBPE at 298 K (a), 308 K (b), 318 K (c), and on SBPEAC at (d) 298 K, (e) 308 K, (f) 318 K.
Figure S3	Non-linear kinetic models for MG adsorption on SBPE (a), and SBPEAC (b) composites.
Figure S4	Van’t Hoff plots for MG adsorption on SBPE (a), and SBPEAC (b) composites.

Text S1.

Non-linear forms of Langmuir [63] (Equation (S1)) and Freundlich [64] (Equation (S2)), and Dubinin–Radushkevich (D-R) (Equations (S3) – (S5)) [65] isotherm models were applied to analyze the experimental data.

$$q_e = \frac{q_m K_L C_e}{1 + K_L C_e} \tag{S1}$$

$$q_e = K_F \, C_e^{1/n} \tag{S2}$$

$$q_e = \; q_s \, e^{-K_{DR} \, \varepsilon^2} \tag{S3}$$

$$\varepsilon = RTln(1 + \frac{1}{C_e} \;) \tag{S4}$$

$$E = \frac{1}{\sqrt{2K_{DR}}} \tag{S5}$$

where, K_L (L/mg), K_F (mg/g)(L/mg)$^{1/n}$, and n are Langmuir constant, Freundlich constant, and degree of the adsorption process, respectively. C_e (mg/L) is the MG concentration at equilibrium, q_e (mg/g) is the amount of MG adsorbed onto SBPE and SBPEAC composites at equilibrium; Q_m (mg/g) is the maximum adsorption capacity; q_s (mg/g) is the adsorption capacity; K_{DR} (mol^2/kJ^2) is the constant related to the sorption energy; ε is the Polanyi potential; and E (kJ/mol) is the mean adsorption energy.

Text S2.

Non-linear pseudo-first order [69] (Equation (S6)), pseudo-second-order (Equation (S7)) [69], and Elovich models (Equation (S8)) [70] were used to investigate the reaction mechanism and rate of MG adsorption on SBPE and SBPEAC composites.

$$q_t = q_e(1 - e^{-K_1 t}) \quad \text{(S6)}$$

$$q_t = \frac{q_2^2 k_2 t}{1 + q_e K_2 \ t} \quad \text{(S7)}$$

$$q_t = \frac{1}{\beta} \ln(1 + \alpha\beta t) \quad \text{(S8)}$$

where, k_1 (1/min), and k_2 (g/mg min) are the rate constants for pseudo- first-order and pseudo-second-order models, respectively; q_t and q_e are the amounts of MG adsorbed at time t and equilibrium, respectively; α (mg/g-min) is the initial adsorption rate; β (mg/g) is the desorption constant during any one experiment.

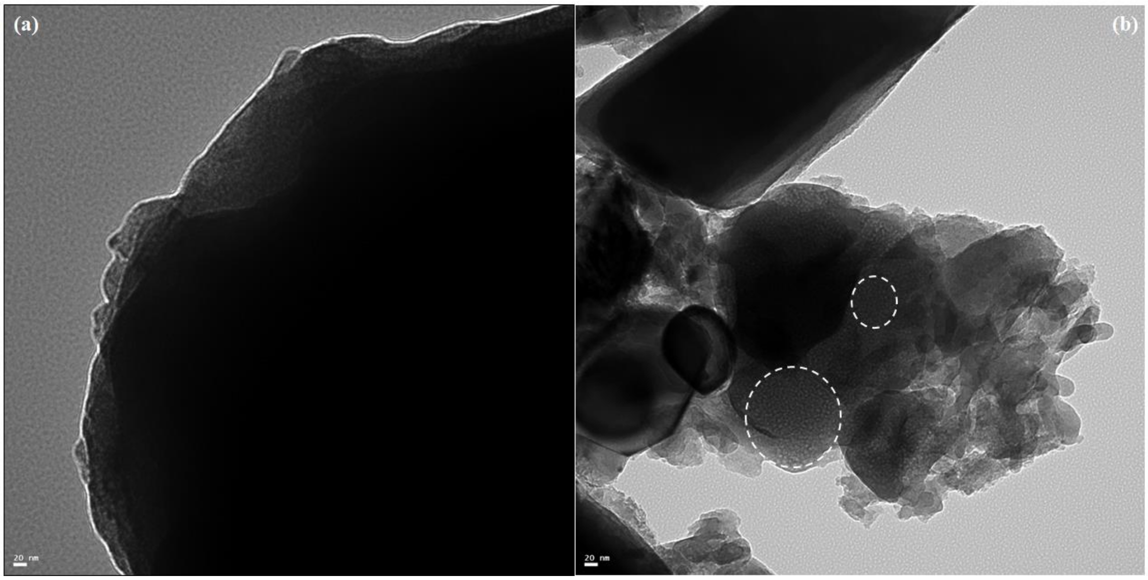

Figure S1. TEM images of SBPE (a), and SBPEAC (b) composites.

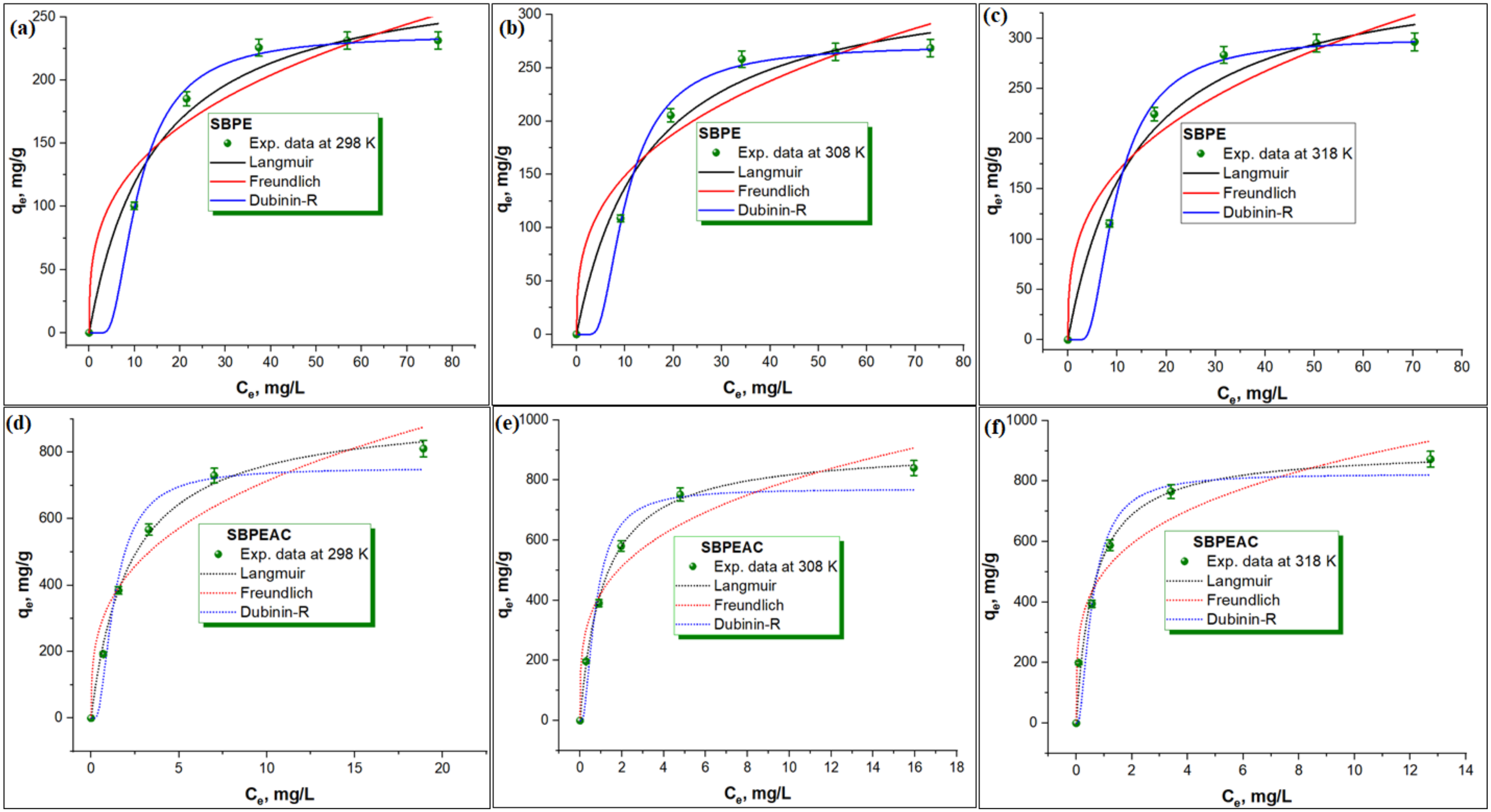

Figure S2. Non-linear isotherm models for MG adsorption on SBPE at 298 K (a), 308 K (b), 318 K (c), and on SBPEAC at (d) 298 K, (e) 308 K, (f) 318 K.

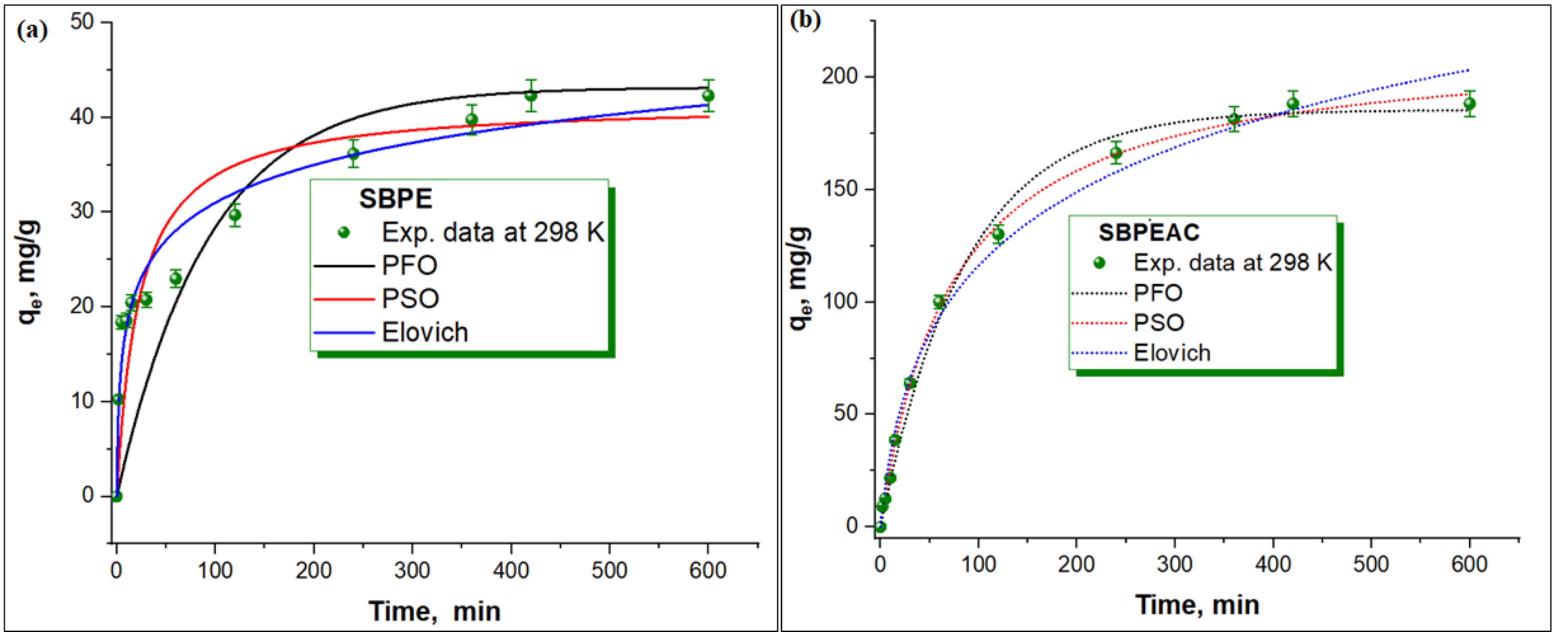

Figure S3. Non-linear kinetic models for MG adsorption on SBPE (a), and SBPEAC (b) composites.

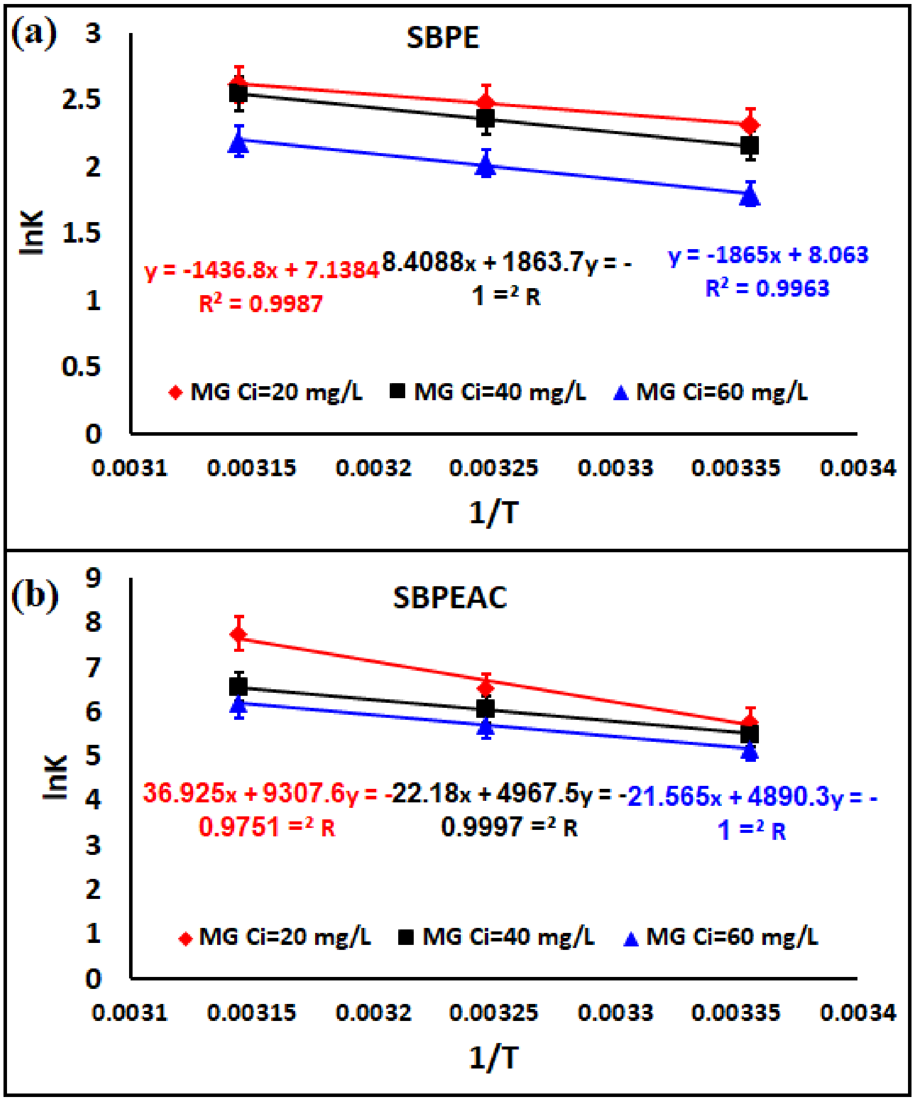

Figure S4. Van’t Hoff plots for MG adsorption on SBPE (a), and SBPEAC (b) composites.

References

63. Wallis ,A.; Dollard, M.F. Local and global factors in work stress—The Australian dairy farming exemplar. *Scand. J. Work. Environ. Health Suppl.* **2008**, *34*, 66–74.
64. Freundlich, H. Über die Adsorption in Lösungen. *Zeitschrift Phys. Chemie* **1907**, *57U*, 385–470. https://doi.org/10.1515/zpch-1907-5723.
65. Dubinin, M.M. The Equation of the Characteristic Curve of Activated Charcoal. *Proc. Acad. Sci. Phys. Chem. Sect.* **1947**, *55*, 331.
69. Lagergren, S. About the theory of so-called adsorption of soluble substances. *Handlingar* **1898**, *24*, 1–39.
70. Chien, S.H.; Clayton, W.R. The catalytic oxidation of carbon monoxide on manganese dioxide. *Sci. Soc. Am. J.* **1980**, *44*, 265–268.
